# Synergistic potential in spinel ferrite MFe_2_O_4_ (M = Co, Ni) nanoparticles-mediated graphene oxide: Structural aspects, photocatalytic, and kinetic studies

**DOI:** 10.1038/s41598-024-55452-4

**Published:** 2024-02-26

**Authors:** Mahnaz Parastar Gharehlar, Shabnam Sheshmani, Farrokh Roya Nikmaram, Zohreh Doroudi

**Affiliations:** Department of Chemistry, College of Basic Sciences, Yadegar-e-Imam Khomeini (RAH) Shahre Rey Branch, Islamic Azad University, Tehran, Iran

**Keywords:** Spinel ferrite nanoparticles, Graphene oxide matrix, Photodegradation, Organic dye pollutants, Textile wastewater, Biogeochemistry, Environmental sciences

## Abstract

The existence of artificial dyes in water is a significant environmental concern, as it can lead to poor water quality. Photodegradation is becoming an increasingly popular method for treating water contaminated with dyes. In this study, the photodegradation of Reactive Red 66 and Reactive Red 120 dyes, as well as textile wastewater, was investigated under UV and visible light irradiation. To enhance the photoresponse of the MFe_2_O_4_ (M = Co, Ni) nanoparticles, modifications were made by incorporating graphene oxide. The MFe_2_O_4_ nanoparticles and MFe_2_O_4_/GO nanocomposite photocatalysts were subjected to several characterization techniques, including FT-IR, Raman spectroscopy, XRD, DRS, zeta potential, VSM, TGA, DSC, BET, SEM, and EDAX analysis. Experiments were conducted to optimize several key parameters involved in the photodegradation process, including pH, photocatalyst dosage, initial dye concentration, and irradiation time. The removal efficiency of Reactive Red 66 and Reactive Red 120 dyes using CoFe_2_O_4_ nanoparticles was found to be 86.97 and 82.63%, respectively. Also, the removal percentage of these dyes using CoFe_2_O_4_/GO nanocomposite photocatalyst was 95.57 and 90.9% for Reactive Red 66 and Reactive Red 120, respectively. Experiments found that NiFe_2_O_4_ nanoparticles removed 90.92% of Reactive Red 66 dye and 84.7% of Reactive Red 120 dye. The NiFe_2_O_4_/GO nanocomposite photocatalyst showed even higher removal efficiencies, degrading 97.96% of Reactive Red 66 and 93.44% of Reactive Red 120. After three days of exposure to visible light irradiation, the removal percentage of Reactive Red 66 using MFe_2_O_4_ and MFe_2_O_4_/GO nanocomposite was investigated.

## Introduction

Spinel ferrites (MFe_2_O_4_) have diverse properties that make them attractive for various applications. They possess excellent magnetic properties, making them essential in magnetic devices like recording devices, data storage, sensors, and electromagnetic interference shielding^1–3^. Spinel ferrites are known for their exceptional electrical conductivity and dielectric properties. They possess desirable electrical characteristics such as high resistivity, low dielectric loss, and excellent insulation properties. These attributes make them suitable for applications in electronics, telecommunications, microwave devices, and magnetic sensors^4,5^. Spinel ferrites exhibit excellent chemical stability, offering resistance to corrosion and oxidation. This inherent stability ensures their durability and reliability in various environments, including harsh and corrosive conditions^6^. Spinel ferrites have a high Curie temperature (Tc), which is the temperature at which a material transitions from ferromagnetic to paramagnetic. They generally have elevated Curie temperatures, allowing them to maintain their magnetic properties even at high temperatures. This makes them valuable in applications that require stable magnetic behavior under high-temperature conditions, such as microwave devices and high-frequency transformers^7^.

Certain modified and engineered spinel ferrites display impressive photocatalytic activity, enabling them to initiate or facilitate chemical reactions when exposed to light. This unique property has led to their investigation for various applications, including photocatalytic water splitting, dye degradation, and environmental remediation^8^. Spinel ferrites have favorable band gap energies, allowing them to absorb UV and/or visible light efficiently. This absorption ability enables spinel ferrites to utilize light energy, creating electron–hole pairs and initiating photocatalytic reactions^9^. Spinel ferrites can be modified or functionalized to enhance their photocatalytic performance, optimizing efficiency and expanding application possibilities. Doping involves introducing impurities or foreign elements into the lattice structure of spinel ferrites, altering their electronic band structure, surface properties, and charge carrier dynamics. Transition metal ions like Co, Ni, Cu, Mn, and Zn can be incorporated through doping in spinel ferrites, significantly influencing their photocatalytic performance. These dopants can modify the band gap energy, increase light absorption, and improve charge separation and transfer efficiency^10^. Consequently, spinel ferrites exhibit significantly enhanced photocatalytic activity, promising them for various photocatalytic applications. Non-metal elements such as nitrogen (N), sulfur (S), carbon (C), and fluorine (F) can be effectively introduced into spinel ferrite photocatalysts. Including non-metal dopants modifies the electronic structure of spinel ferrites, creating additional active sites and improving photocatalytic efficiency and selectivity. Non-metal doping presents a promising strategy to optimize the performance of spinel ferrite photocatalysts for diverse applications^11,12^. The addition of noble metal nanoparticles like gold (Au), silver (Ag), or platinum (Pt) onto spinel ferrite surfaces greatly enhances their photocatalytic properties. These noble metal nanoparticles act as co-catalysts, promoting efficient charge transfer and surface reactions. This synergistic effect leads to a substantial improvement in the photocatalytic performance of spinel ferrites, making them highly effective for diverse photocatalytic applications^13^. Co-doping involves introducing multiple dopants simultaneously into spinel ferrites, combining the benefits of transition metal doping and non-metal doping. This synergistic approach enhances the photocatalytic activity by improving light absorption, modifying the band structure, and enhancing charge separation and migration processes. Co-doping is a powerful method to optimize and improve the overall photocatalytic performance of spinel ferrites. Doping spinel ferrites with heteroatoms like boron (B), phosphorus (P), or iodine (I) introduce new energy levels within the band structure. These heteroatoms create active sites that promote efficient charge transfer and drive photocatalytic reactions. By incorporating heteroatoms, spinel ferrites acquire improved photocatalytic properties, enabling effective light energy utilization for desired chemical transformations. Heteroatom doping presents a promising approach to customize the photocatalytic performance of spinel ferrites to meet specific application needs^14^.

Photocatalysts based on nanoparticles have emerged as a promising chemical method for dye degradation. These nanoparticles have attracted significant interest due to their remarkable properties and wide-ranging potential in environmental remediation, energy conversion, and photocatalysis-driven reactions. Primarily consisting of semiconductors, these nanoparticles exhibit excellent photocatalytic activity attributed to their expansive surface area, effective charge separation, and customizable electronic and optical properties^15–17^.

In recent years, our research group has made significant progress in evaluating a range of photocatalysts for the efficient degradation of dye pollutants in aqueous media and wastewater. To date, there has been limited scientific research on using magnetic spinel ferrite for degrading Reactive Red 66 (R66) and Reactive Red 120 (R120) dyes. The objective of this study is to develop nanoparticles of spinel ferrite (MFe_2_O_4_, M = Co, Ni) and composites of MFe_2_O_4_/graphene oxide that can effectively remove R66 and R120 from aqueous solutions and textile wastewater. The research includes a comprehensive exploration of composite preparation, encompassing morphological and crystallographic characterizations, as well as kinetics, with the primary aim of demonstrating the potential application of these magnetic nanocomposites.

## Materials and methods

### Materials

In this study, ferric chloride (FeCl_3_, 98%, Merck), ferrous chloride tetrahydrate (FeCl_2_∙4H_2_O, 98%, Merck), cobalt chloride hexahydrate (CoCl_2_∙6H_2_O, 98%, Merck), and nickel nitrate hexahydrate (Ni(NO_3_)_2_∙6H_2_O, 98%, Merck) were utilized. Graphite flakes (99.5%, Sigma-Aldrich), sodium nitrate (NaNO_3_, 99.9%, Merck), potassium permanganate (KMnO_4_, 99.5%, Merck), hydrogen peroxide (H_2_O_2_, 30%, Merck), aqueous ammonia (NH_3_(aq), 99.9%, Merck) and sulfuric acid (H_2_SO_4_, 97%, Merck) were also employed in this study. Reactive Red 66 (C_20_H_15_BrN_4_Na_2_O_8_S_2_, R66, a monoazo dye) and Reactive Red 120 (C_44_H_24_Cl_2_N_14_Na_6_O_20_S_6_, R120, diazo dye) were sourced from Nordex International and D.Z.E Dye Company in the UK, respectively.

### Methods

Fourier transform infrared spectroscopy (FT-IR) measurements were conducted using Thermo AVATAR equipment across the 400–4000 cm^−1^ range. This technique is employed to gather information regarding the chemical composition. Vibration modes were determined using a Raman Takram, P50C0R10 TEKSAN apparatus. The excitation source was 532 nm, and the laser power ranged from 0.5 to 70 mW. Powder X-ray diffraction (XRD) patterns were measured using a PHILIPS PW1730 instrument equipped with Cu Kα radiation. The intensity data was collected within a 2θ range of 10 to 80°. The patterns were indexed using the Joint Committee of Powder Diffraction Standard (JCPDS) database files.

Zeta potential measurements were performed using a Zeta Horiba instrument, Zeta-Dls Zetasizer, from Malvern, Cambridge, UK. This technique determines the surface charge of particles in a suspension, providing valuable information about suspension stability and its interaction potential with other surfaces or dyes. The magnetic properties of the samples were studied using a vibrating sample magnetometer (VSM) in the LBKFB 1.4 T equipment. VSM analysis involves measuring the magnetic hysteresis loops of small material samples by varying the strength of the applied magnetic field and observing the resulting magnetization. Diffuse reflectance spectroscopy (DRS) was performed using the SCINCO S-4100 instrument to investigate the optical properties of a material across the ultraviolet, visible, and near-infrared regions of the electromagnetic spectrum. By analyzing the intensity and wavelength of the scattered light, valuable insights can be obtained regarding the electronic structure of the material, including the determination of its band gap energy. The TGA Q600 analysis offers essential supplementary data concerning the thermal stability, composition, and interactions within the prepared nanocomposites. Additionally, the BELSORP Mini II device was employed for Brunauer–Emmett–Teller (BET) analysis to ascertain surface area and average pore diameter.

The morphology and particle size of the samples were examined using the TESCAN MIRA III model, a field emission gun (FEG) electron microscope operating in scanning electron microscope (SEM) mode. To enhance resolution, all samples were gold-sputtered before imaging. The SEM unit was equipped with an Energy Dispersive Spectroscopy (EDS) system, allowing for comprehensive analysis of sample composition at the microscopic level.

In this study, a 400 W mercury vapor lamp was utilized as the light source, emitting a wide range of wavelengths, including ultraviolet (UV) radiation. The lamp was positioned approximately 25 cm away from the sample solution. The dye concentration was measured using UV–Vis spectroscopy with a Cary 60 UV–Vis spectrophotometer from Agilent Technologies (USA). Additionally, the sample was exposed to laboratory sunlight, which served as a natural source of light irradiation. Sunlight encompasses a broad spectrum of wavelengths, including ultraviolet (UV), visible (V), and infrared (IR) light. By subjecting the sample to sunlight, it undergoes interactions with this natural light source.

### Preparation of spinel ferrite nanoparticles (MFe_2_O_4_, M = Co, Ni)

This procedure was performed to prepare the CoFe_2_O_4_ nanoparticles. Iron(III) chloride hexahydrate (2 mmol in 15 mL water) and cobalt(II) chloride hexahydrate (1 mmol in 15 mL water) were accurately measured and mixed. Drop by drop, ammonium hydroxide solution (25%) was added to the mixture until the pH reached 4. Additional ammonium hydroxide solution (25%) was added until the pH reached 12. The resulting mixture was stirred for 30 min, ensuring thorough mixing. The mixture was then transferred to a Teflon autoclave and placed in an oven set at a temperature of 180 °C. It was allowed to react for 13 h. Once the reaction time elapsed, the autoclave was cooled down to room temperature. The precipitates formed were separated by filtration and washed multiple times using water and ethanol. The washing process continued until the pH of the filtrate reached 7. The filtered composite was carefully dried in a vacuum oven at 60 °C for 12 h.

The method for preparing NiFe_2_O_4_ is essentially the same as the method for preparing CoFe_2_O_4_, with the only difference being the substitution of CoCl_2_∙6H_2_O with Ni(NO_3_)_2_∙6H_2_O.

Finally, the spinel ferrite MFe_2_O_4_ (M = Co, Ni) was subjected to various analytical techniques for identification and characterization.

### Preparation of graphene oxide (GO)

To synthesize graphene oxide, a reaction vessel was used to combine graphite flakes (3 g) and sodium nitrate (3 g). The vessel was then placed in an ice bath to create a controlled low-temperature environment. Over, one hour, sulfuric acid (20%, 150 mL) was slowly and thoroughly added to the mixture. Following that, potassium permanganate (9 g) was introduced. The reaction vessel was heated to 40 °C and stirred for 2 h until the mixture obtained a waxy consistency.

Afterward, distilled water (150 mL) was added to the reaction mixture, and the temperature was raised to approximately 90 °C. It was maintained at this level for 30 min during the reaction. While stirring, hydrogen peroxide (20%, 30 mL) was gradually incorporated into the mixture, resulting in a color change to a dark brown hue.

Once the reaction was complete, the mixture was centrifuged to separate the graphene oxide. The separated graphene oxide was washed with deionized water until it reached a neutral pH. Finally, the graphene oxide was dried in an oven at a moderate temperature (60 °C) for 12 h.

### Preparation of MFe_2_O_4_/graphene oxide (MFe_2_O_4_/GO, M = Co, Ni)

The preparation method for MFe_2_O_4_/GO follows a similar procedure to the synthesis of MFe_2_O_4_ nanoparticles. In this method, Fe^3+^ and Co^2+^ precursors, along with NH_3_, are transferred to an autoclave. Additionally, a graphene oxide solution consisting of 0.1 g of graphene oxide dissolved in 40 mL of distilled water is prepared. The graphene oxide solution undergoes ultrasound treatment for 1 h to ensure proper dispersion. After the ultrasound treatment, the graphene oxide solution is carefully and gradually added to a pre-prepared mixture within the autoclave and placed in an oven set at 180 °C for 13 h.

### Photocatalysis studies

Photodegradation experiments were performed to assess the efficacy of photocatalyst nanocomposites in the degradation of Reactive Red 66 (λmax = 516 nm) and Reactive Red 120 dyes (λmax = 537 nm) dyes. The experiments involved using a dye solution with a concentration of 20 mg/L and a volume of 25 mL at a temperature of 25 °C. To achieve significant dye removal, 0.05 g of the nanocomposites were added to the solution and vigorously stirred at 150 rpm for 30 min at various pH levels. The mixture was then separated using a magnet, and the concentration of the dye was measured. Dye removal experiments were conducted by altering the initial pH level (ranging from 2 to 9), the initial dye concentration (ranging from 5 to 50 mg/L), the dosage of the photocatalyst (ranging from 0.01 to 0.08 g), and the duration of UV irradiation (ranging from 15 to 90 min). Furthermore, degradation studies were also carried out under sun light irradiation in order to assess the efficacy of the photocatalyst composites under natural sun light conditions. The dye solutions were exposed to direct sunlight for three days. Control experiments were also performed by irradiating dye solutions without any added photocatalyst under identical conditions, but no dye degradation was observed in the absence of the photocatalyst nanocomposites.

## Results and discussions

Spinel ferrites, with the general chemical formula MFe_2_O_4_, feature a structure where iron (Fe) ions occupy tetrahedral sites and divalent metal cations like cobalt (Co), nickel (Ni), zinc (Zn), or manganese (Mn) occupy octahedral sites. These sites are part of a face-centered cubic (FCC) lattice arrangement of oxygen ions. Within this structure, the octahedral sites are coordinated by six oxygen ions, forming an octahedral coordination environment. In comparison, the tetrahedral sites are coordinated by four oxygen ions, creating a tetrahedral coordination environment. The arrangement of metal cations within the spinel structure forms an interconnected network of octahedral and tetrahedral sites exhibiting unique properties.

The spinel ferrite structure offers various advantages for different applications. It allows for including different metal cations, enabling the tuning of properties. Moreover, the presence of diverse coordination environments within both the tetrahedral and octahedral sites accommodates other ions and functional groups. These characteristics contribute to the intriguing properties of spinel ferrites, including magnetic, electrical, and catalytic behavior.

### FT-IR studies

**Figure 1:**
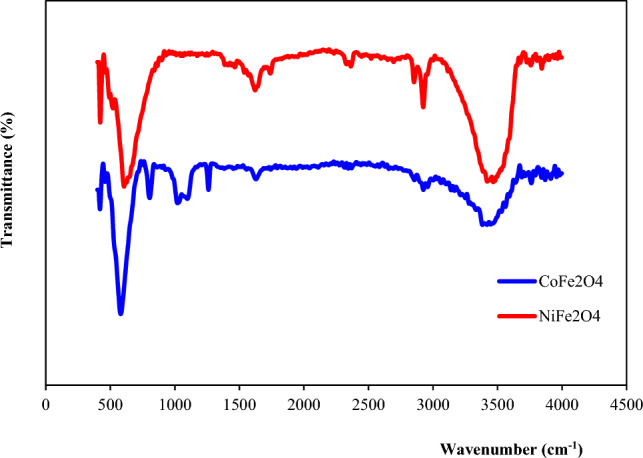
FT-IR spectra of MFe_2_O_4_ (M = Co, Ni).

The Fourier transform infrared (FT-IR) spectra provide evidence for the successful preparation of the CoFe_2_O_4_ and NiFe_2_O_4_ compounds based on the characteristic peaks observed (Fig. 1). In the CoFe_2_O_4_ spectrum, the prominent peak at 580 cm^−1^ corresponds to the Co–O bond stretch, while the peak at 400 cm^−1^ is attributed to the Fe–O bond vibration. Additionally, the broad band spanning 3300–3500 cm^−1^ arises from the O–H stretch of hydroxyl groups on the oxide surface. A distinct peak at 1630 cm^−1^ represents the bending mode of adsorbed water. Similarly, the NiFe_2_O_4_ spectrum displays a peak at 606 cm^−1^ corresponding to the Ni–O vibration and a peak at 421 cm^−1^ associated with the Fe–O vibration, confirming the presence of Ni and Fe oxide bonds in this sample ^18^. The IR spectra thus verify the chemical structure of the prepared CoFe_2_O_4_, and NiFe_2_O_4_ and the signature peaks can serve as an essential benchmark for future photocatalyst preparations.

**Figure 2:**
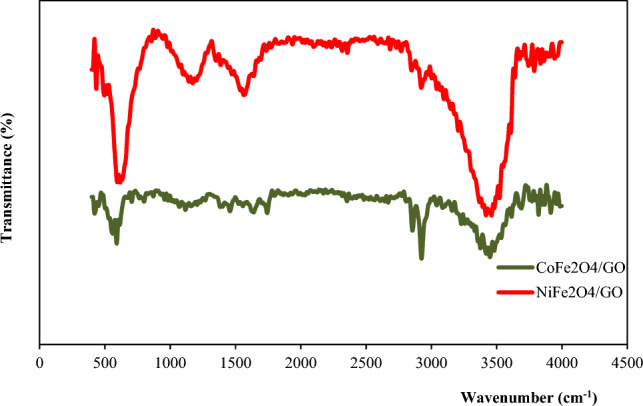
FT-IR spectra of MFe_2_O_4_/GO (M = Co, Ni).

In addition to the presence of ferrite-related functional groups, the IR spectrum of MFe_2_O_4_/GO (M = Co, Ni) compounds also reveals vibrations characteristic of graphene oxide (Fig. 2). Specifically, the C–O stretching vibration, found within the range of 1000–1200 cm^−1^, corresponds to the stretching of carbon–oxygen (C–O) bonds present in functional groups such as hydroxyl (–OH), epoxy (–O–), and carboxyl (–COOH) groups. The C=O stretching vibration typically observed around 1700 cm^−1^, signifies the presence of carbonyl groups (–C=O) within the graphene oxide structure. Additionally, the C–C stretching vibration occurring between 1400 and 1650 cm^−1^ is associated with the stretching of carbon–carbon (C–C) bonds within the graphene framework.

### Raman studies

**Figure 3:**
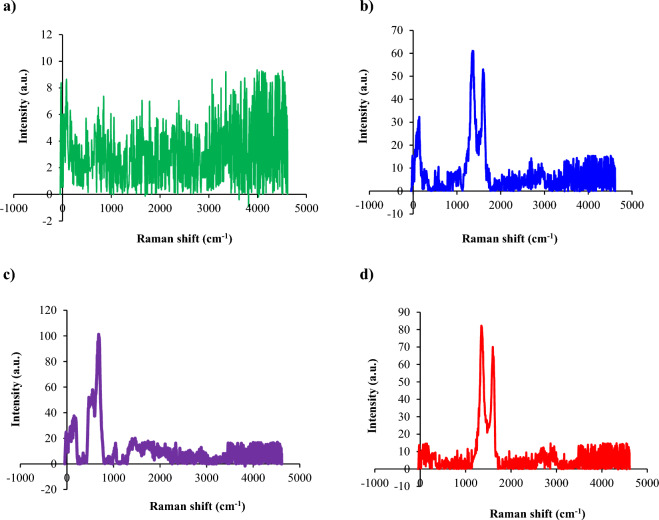
Raman spectra of (**a**) CoFe_2_O_4_, (**b**) CoFe_2_O_4_/GO, (**c**) NiFe_2_O_4_, and (**d**) NiFe_2_O_4_/GO.

In the Raman spectra of CoFe_2_O_4_, multiple distinct vibration modes are observed due to its complex spinel structure. The spinel structure contains tetrahedral and octahedral cation sites occupied by Co^2+^ and Fe^3+^, leading to different metal–oxygen vibrations. The Co–O and Fe–O bonds exhibit peaks at different frequencies owing to variations in bond strength and atomic masses between Co and Fe. Raman selection rules enable observing modes with different symmetries based on the crystal structure symmetry ^19^. The combination of these factors results in the presence of several characteristic peaks corresponding to different vibration modes in the Raman spectra of CoFe_2_O_4_ (Fig. 3a). The incorporation of graphene oxide into the CoFe_2_O_4_ sharpens the peaks observed in the Raman spectrum, making them more distinct from one another. The graphene oxide seems to modify the material in a way that allows the vibration modes to become better defined. This sharpening of the peaks suggests that the addition of graphene oxide induces changes in crystallinity, particle size, or lattice dynamics (Fig. 3b). In the Raman spectra of CoFe_2_O_4_/GO, characteristic peaks corresponding to the vibrational modes of both CoFe_2_O_4_ and graphene oxide is expected to be observed. These peaks include the A_1g_, E_g_, and T_2g_ modes associated with the lattice vibrations of CoFe_2_O_4_. The A_1g_ mode, representing the symmetric stretching vibrations of oxygen atoms in the CoFe_2_O_4_ lattice, is typically observed around 198 cm^−1^. The E_g_ mode, approximately 375 cm^−1^, corresponds to the doubly degenerate vibrations associated with the oxygen octahedral distortion within the CoFe_2_O_4_ crystal structure. Furthermore, the T_2g_ mode, typically observed around 525 cm^−1^, represents vibrations related to the metal–oxygen bonds present in CoFe_2_O_4_ (Fig. 3b). On the other hand, graphene oxide, a layered material, exhibits specific peaks in its Raman spectrum that are directly related to its structure. The most notable peaks include the D band and G band. The D band, typically observed around 1340 cm^−1^, arises from defects and disorder in the graphene lattice. It represents the breathing modes of carbon atoms in graphene oxide. The G band appears at approximately 1588 cm^−1^ and corresponds to the sp^2^ carbon–carbon stretching vibrations in graphene oxide (Fig. 3b).

The NiFe_2_O_4_ has an inverse spinel structure with Ni^2+^ ions on octahedral sites and Fe^3+^ ions occupying both tetrahedral and octahedral sites. Several distinct peaks are observed in the Raman spectra, corresponding to different vibrational modes of the crystal lattice. The main peaks are seen in the range of 400–700 cm^−1^ and are assigned to metal–oxygen stretching and bending modes. The peaks around 670–690 and 550–610 cm^−1^ are attributed to Ni–O and Fe–O vibrations, respectively, in the octahedral sites (Fig. 3c). In the NiFe_2_O_4_/GO raman spectra, the G band around 1580 and D band around 1350 cm^−1^ arise from the sp^2^-bonded carbon structure of graphene oxide. The G band is related to the E_2g_ phonon mode of C–C bonding, while the D band involves phonon-defect scattering. At higher graphene oxide loadings, the NiFe_2_O_4_ vibrational peaks diminish in intensity while the composite shows predominantly graphene oxide bands. The graphene oxide peaks exhibit shifts and broadening, indicating strong interaction with the NiFe_2_O_4_ matrix. The interface between the nickel ferrite and graphene oxide allows tuning properties like conductivity, surface area, and magnetic behavior. This is attributed to the interaction between the two components (Fig. 3d).

### Zeta potential studies

Zeta potential is a vital parameter that reveals the surface charge of particles in a liquid medium, like a colloidal dispersion. It plays a significant role in comprehending the interactions and stability of colloidal systems by indicating the potential for particle attraction or repulsion.

**Figure 4:**
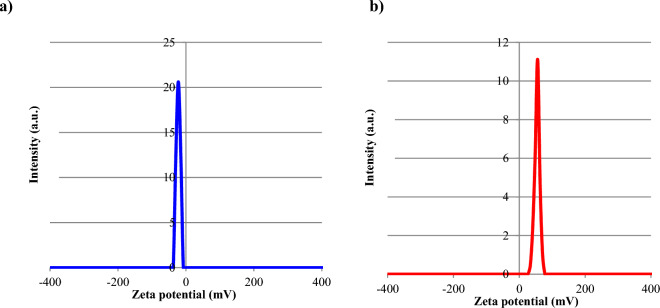
Zeta potential of (**a**) CoFe_2_O_4_/GO and (**b**) NiFe_2_O_4_/GO.

The negative zeta potential observed for CoFe_2_O_4_/GO composite particles can be attributed to several factors. While pristine CoFe_2_O_4_ has an electrically neutral surface, dispersion in an aqueous medium enables surface interactions with ions that can impart charge. However, the predominant source of negative charge likely arises from the graphene oxide (GO) component. GO contains abundant oxygen functional groups like carboxyl's and hydroxyls that ionize in solution to produce a negative surface charge. Therefore, the overall negative zeta potential of the CoFe_2_O_4_/GO composite stems mainly from the charged oxygen moieties on the GO sheets. This negative surface charge critically impact the colloidal stability and interactions of the CoFe_2_O_4_/GO particles. The mutual electrostatic repulsion between particles imposed by the negative zeta potential hinders particle aggregation. This electrostatic stabilization mechanism enables the uniform dispersion of the composite nanoparticles and prevents their flocculation and precipitation from the colloidal medium (Fig. 4a).

The positive zeta potential observed for NiFe_2_O_4_/GO composite particles indicates a positive charge on their surfaces. Unlike pristine NiFe_2_O_4_ with a near neutral surface charge, the positive zeta potential arises from charged species adsorbed from the surrounding medium. Specifically, dissolved metal cations are electrostatically attracted to the surface, imparting a positive charge. The graphene oxide (GO) sheets likely provide favorable sites for cation adsorption due to their oxygen functional groups. This positive surface charge has important implications for the colloidal stability and interactions of the NiFe_2_O_4_/GO particles. The mutually repulsive forces between particles imposed by their positive zeta potential prevent aggregation. Additionally, the positively charged composite surface influences interactions with other charged species present in solution or at interfaces. This charge-dependent behavior can be leveraged for directed assembly or to promote adhesion to negatively charged surfaces. Overall, the positive zeta potential of NiFe_2_O_4_/GO particles provides electrostatic stabilization and enables charge-based control over the composite's interactions in solution (Fig. 4b).

### VSM studies

**Figure 5:**
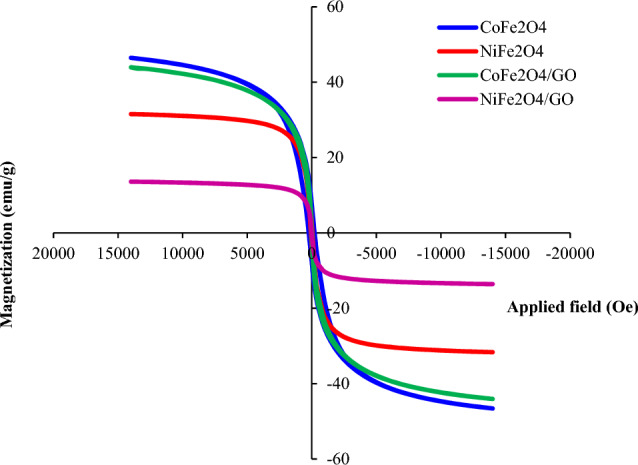
VSM analysis of MFe_2_O_4_ and MFe_2_O_4_/GO (M = Co, Ni).

According to the VSM analysis, both CoFe_2_O_4_ and CoFe_2_O_4_/GO demonstrate similar magnetization values of approximately 40 emu (Fig. 5). This similarity suggests that these materials possess comparable magnetic properties at the specific measurement point. The presence of graphene oxide in the CoFe_2_O_4_/GO composite does not significantly affect the overall magnetization, suggesting that it has a minimal impact on the magnetic behavior of cobalt ferrite. In contrast, the VSM measurements of NiFe_2_O_4_ and NiFe_2_O_4_/GO reveal different magnetization values (Fig. 5). NiFe_2_O_4_ exhibits a magnetization value of approximately 25 emu, whereas NiFe_2_O_4_/GO shows a lower value of 10 emu. This difference suggests that the incorporation of graphene oxide has influenced the magnetic properties of the NiFe_2_O_4_ composite. GO appears to have reduced the overall magnetization, resulting in a lower magnetic moment compared to pure NiFe_2_O_4_. As can be seen, magnetite and its composites show magnetism saturation with superparamagnetic nature^17^.

### XRD studies

**Figure 6:**
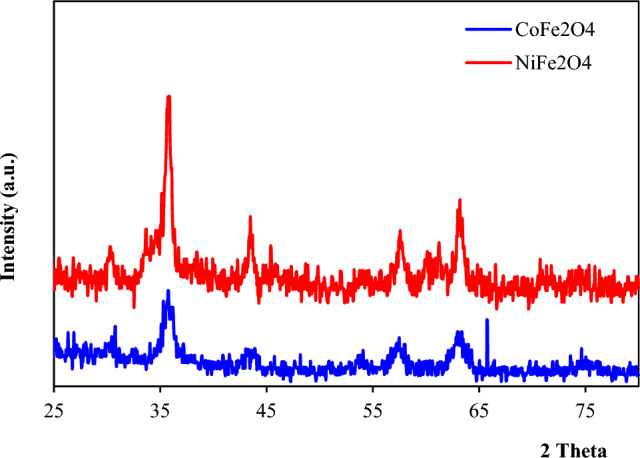
XRD pattern of MFe_2_O_4_ (M = Co, Ni).

The crystalline phases of the prepared cobalt ferrite (CoFe_2_O_4_) and nickel ferrite (NiFe_2_O_4_) photocatalysts were analyzed by X-ray diffraction (XRD)^19^. The XRD patterns contain distinct characteristic peaks corresponding to the specific lattice planes in the crystal structure of the material. The diffractogram of CoFe_2_O_4_ (Fig. 6) displays intense peaks at 2θ values of 30.7, 35.7, 42.6, 57.1, and 62.8°, indexed to the (220), (311), (400), (511), and (440) planes of the spinel cobalt ferrite structure (JCPDS 22-1086). The presence of these planes confirms the successful formation of highly crystalline CoFe_2_O_4_. Similarly, the NiFe_2_O_4_ photocatalyst may exhibit prominent XRD peaks around 2θ values of 30.2, 35.6, 43.4, 57.4, and 63.1º, corresponding to the (220), (311), (400), (511), and (440) crystallographic planes. Observing these peaks at specific positions signifies the development of the signature spinel structure of nickel ferrite.

### DRS studies

**Figure 7:**
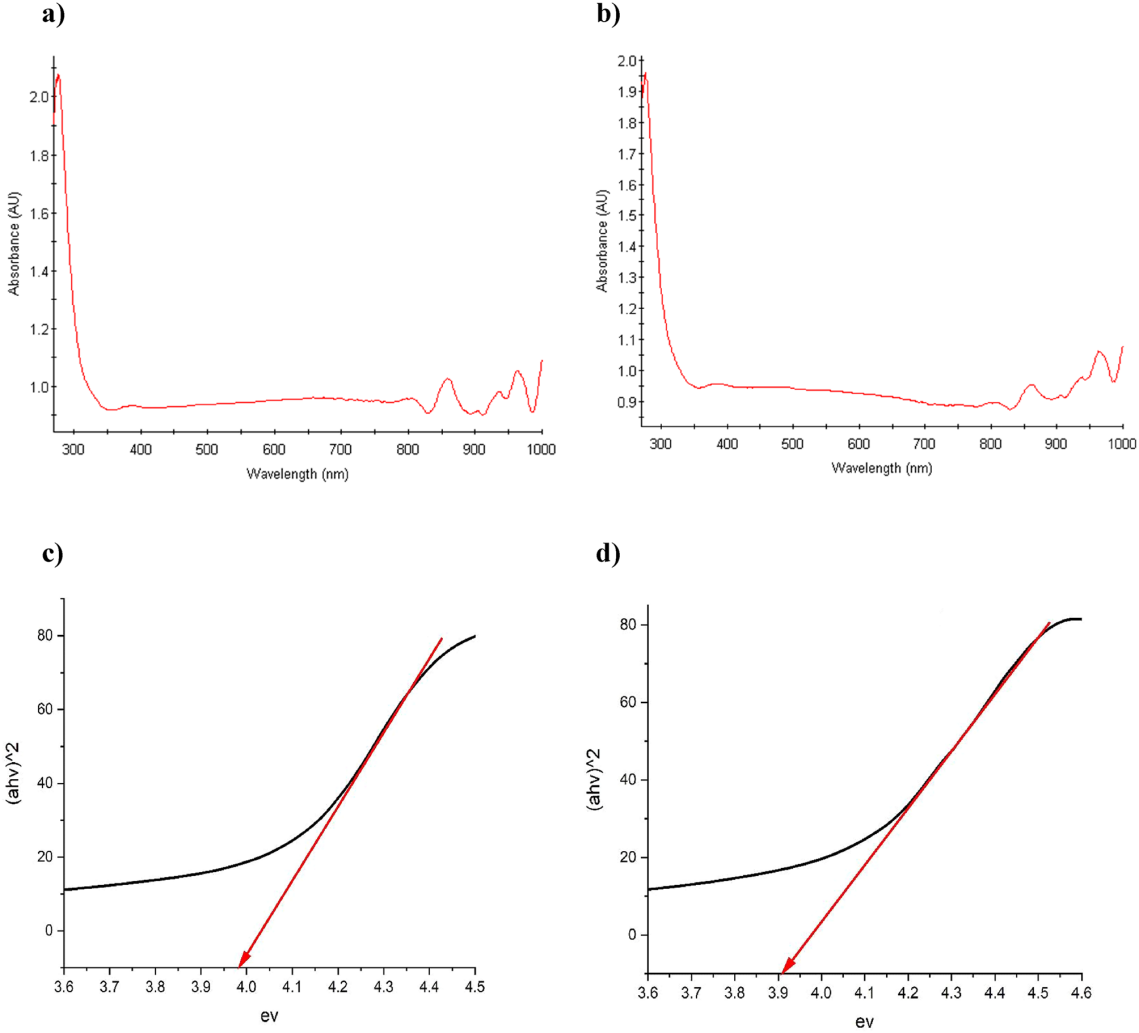
Absorbance spectra of (**a**) CoFe_2_O_4_, (**b**) NiFe_2_O_4_, and band gap values obtained for (**c**) CoFe_2_O_4_, (**d**) NiFe_2_O_4_ using DRS.

**Figure 8:**
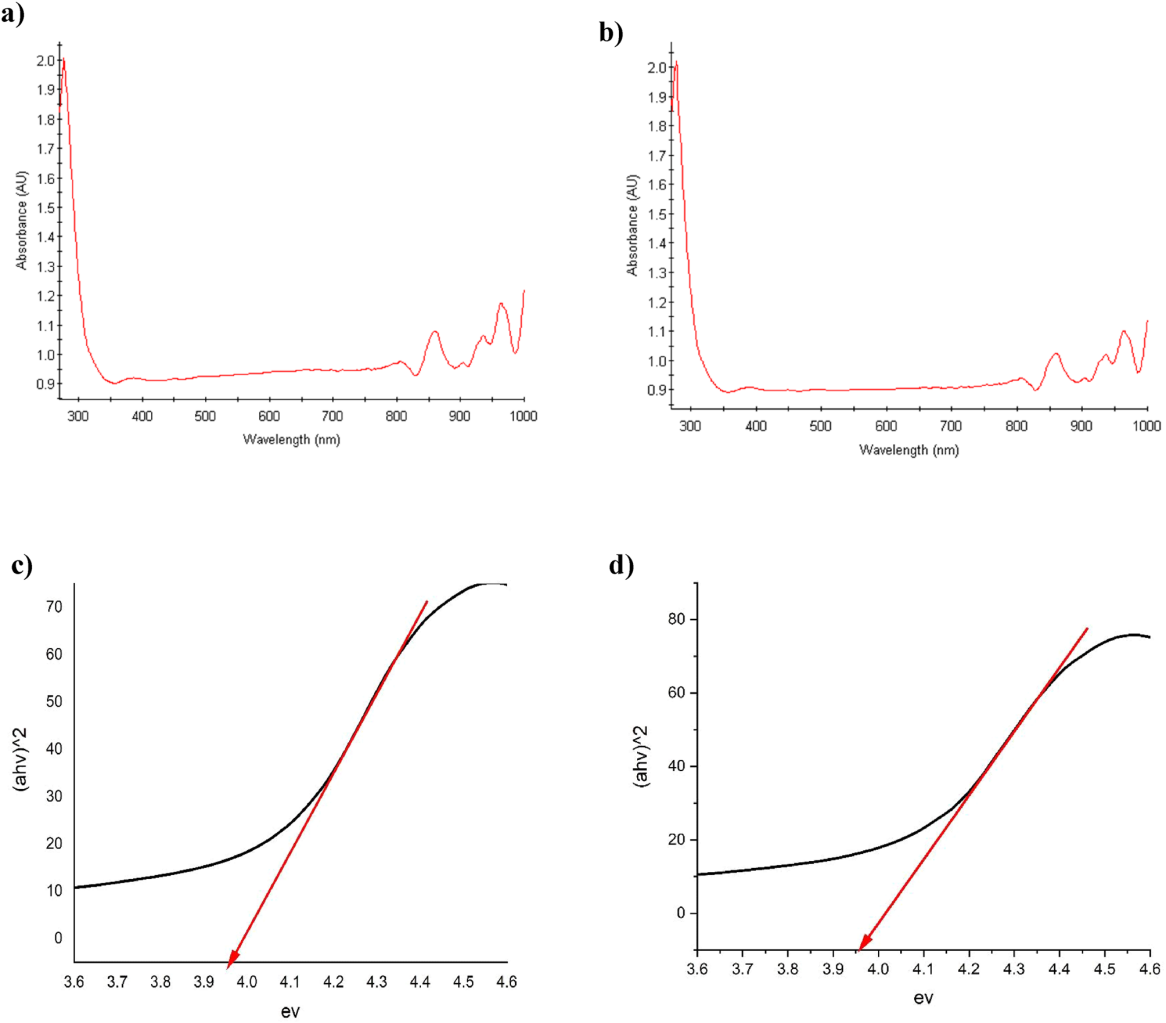
Absorbance spectra of (**a**) CoFe_2_O_4_/GO, (**b**) NiFe_2_O_4_/GO, and band gap values obtained for (**c**) CoFe_2_O_4_/GO, (**d**) NiFe_2_O_4_/GO using DRS.

Diffuse reflectance spectroscopy (DRS) was utilized to investigate the band gap of the MFe_2_O_4_ material and the MFe_2_O_4_/GO composite (Figs. 7, 8). Through analysis of the absorbance spectra obtained from the DRS measurements, the band gap values could be estimated. The observed absorption edges in the spectra indicated the initiation of electronic transitions within both the material and its composite^20,21^. The technique measures the diffused reflectance intensity as a function of incident photon wavelength. Analysis of the DRS spectrum enables the determination of the adequate optical band gap energy (Eg) based on the Tauc plot method. In this approach, the absorption coefficient (α) is plotted against the photon energy (hv) and extrapolated to α = 0 to estimate Eg. An assumption of a direct allowed electronic transition is made, and (αhv)^2^ *vs.* hv is plotted according to Tauc's formula. CoFe_2_O_4_ and NiFe_2_O_4_ nanoparticles have established band gap values of 3.98 eV and 3.90 eV, respectively, signifying their classification as semiconductors with relatively wide band gaps. A larger band gap implies that these compounds require higher energy, in the form of photons, to facilitate the movement of electrons from the valence band to the conduction band. Consistently, the band gap values for both CoFe_2_O_4_/GO and NiFe_2_O_4_/GO are reported as 3.95 eV. Notably, the band gap values for CoFe_2_O_4_ and NiFe_2_O_4_ nanoparticles remain unchanged in the MFe_2_O_4_/GO composite, suggesting that the incorporation of graphene oxide (GO) did not significantly alter the electronic structure or introduce new electronic states that would affect the composite's band gap. This indicates that the electronic properties of the composite are primarily governed by the individual properties of the MFe_2_O_4_ nanoparticles.

### SEM studies

**Figure 9:**
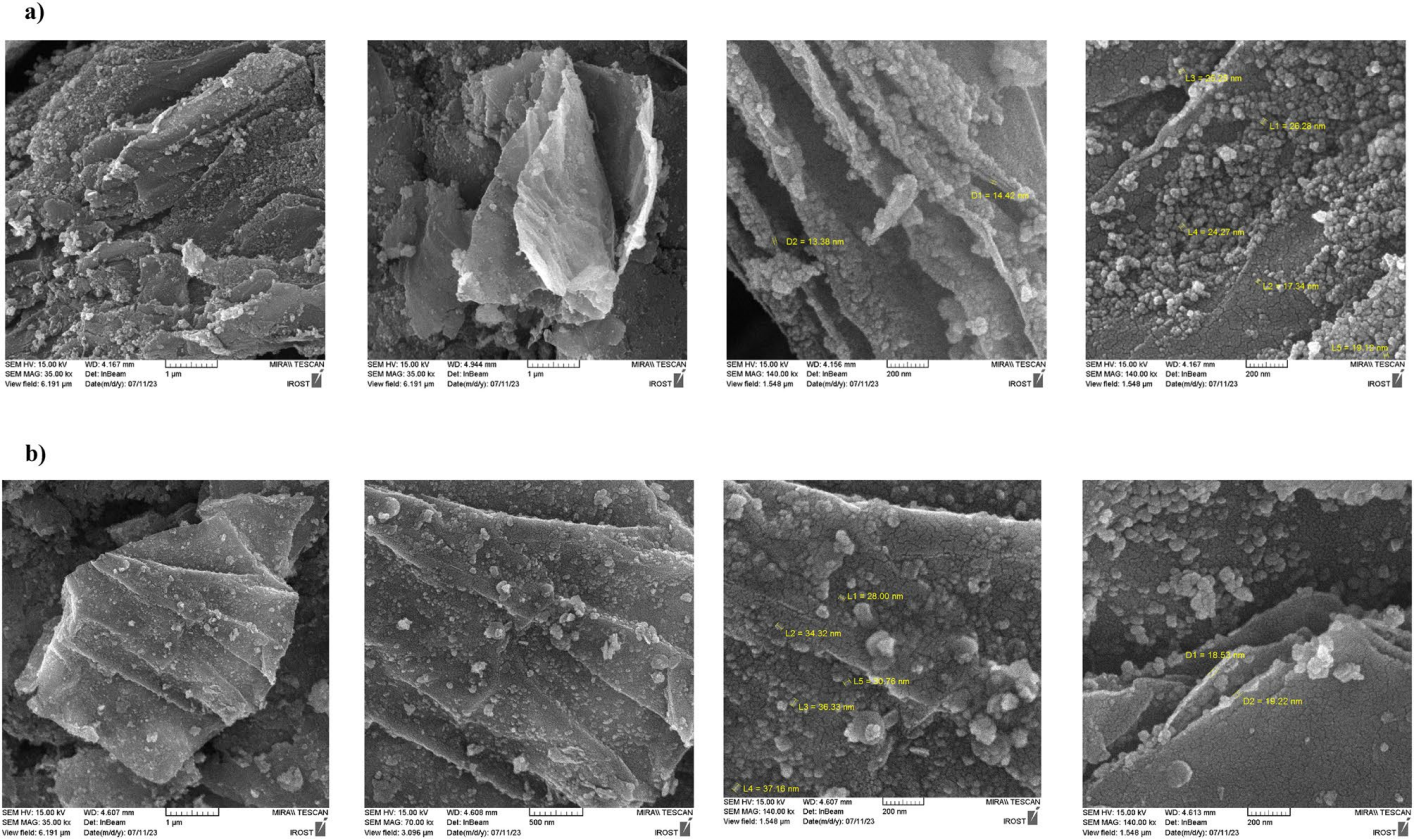
SEM images of (**a**) CoFe_2_O_4_/GO and (**b**) NiFe_2_O_4_/GO.

SEM analysis of the MFe_2_O_4_/GO (M = Co, Ni) composite can provide visual information about the morphology and microstructure, including the size and spatial arrangement of the particles within the graphene oxide matrix^22,23^. In the context of the CoFe_2_O_4_/GO composite, it appears that graphene oxide is used as a supporting matrix for the CoFe_2_O_4_ particles. Graphene oxide is derived from graphite through the introduction of oxygen-containing functional groups. The CoFe_2_O_4_ and NiFe_2_O_4_ particles within the composite have a size range of 14–30 and 19–37 nm, respectively. These particles are likely dispersed or embedded within the graphene oxide sheets, forming a composite structure (Fig. 9). Combining the two materials can result in synergistic properties, leveraging the unique characteristics of both graphene and spinel ferrite of MFe_2_O_4_.

**Figure 10:**
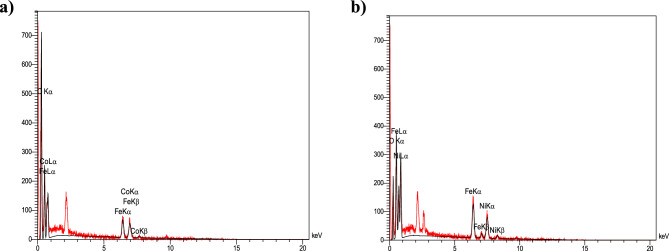
EDS analysis of (**a**) CoFe_2_O_4_/GO and (**b**) NiFe_2_O_4_/GO.

Table 1 presents the elemental composition of the CoFe_2_O_4_/GO nanocomposite. The EDX analysis confirms the presence of carbon (58.75%), oxygen (25.69%), iron (7.64%), and cobalt (7.92%) in the nanocomposite sample. Similarly, for NiFe_2_O_4_, the composition comprises carbon (29.03%), oxygen (32.72%), iron (18.50%), and nickel (19.75%), as shown in Table 2. These results provide validation that the nanocomposite consists of graphene oxide and MFe_2_O_4_ components without detecting any unexpected impurities. Also, Fig. 10 is shown the results of EDX analysis.

**Table 1:** Energy dispersive X-ray spectroscopy (EDA) analyses of CoFe_2_O_4_/GO.

Elt	Line	Int	K	Kr	W%	A%	ZAF	Pk/Bg	LConf	HConf
C	Ka	156.1	0.6399	0.3177	58.75	72.27	0.5407	657.39	57.04	60.47
O	Ka	58.0	0.1184	0.0588	25.69	23.72	0.2288	60.73	24.46	26.92
Fe	Ka	34.0	0.1208	0.0600	7.64	2.02	0.7851	10.23	7.16	8.11
Co	Ka	27.6	0.1209	0.0600	7.92	1.99	0.7579	9.16	7.37	8.47
			1.0000	0.4965	100.00	100.00

**Table 2:** Energy dispersive X-ray spectroscopy (EDA) analyses of NiFe_2_O_4_/GO.

Elt	Line	Int	K	Kr	W%	A%	ZAF	Pk/Bg	LConf	HConf
C	Ka	36.4	0.1793	0.0989	29.03	47.11	0.3408	59.88	27.27	30.78
O	Ka	91.9	0.2257	0.1246	32.72	39.87	0.3807	43.28	31.47	33.96
Fe	Ka	70.0	0.2989	0.1650	18.50	6.46	0.8915	16.16	17.70	19.31
Ni	Ka	44.4	0.2961	0.1634	19.75	6.56	0.8274	12.87	18.67	20.84
			1.0000	0.5519	100.00	100.00

**Figure 11:**
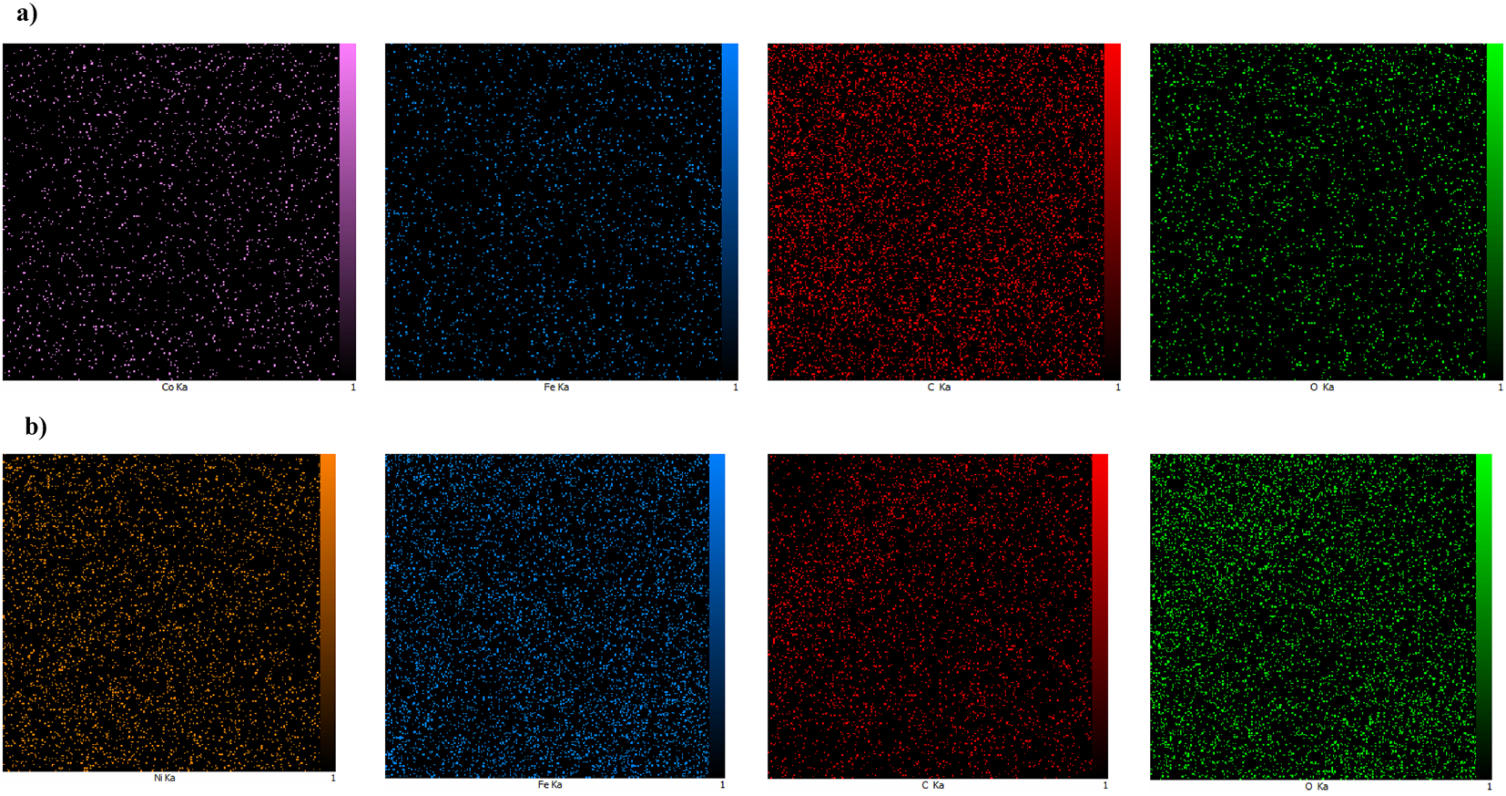
Mapping images of (**a**) CoFe_2_O_4_/GO and (**b**) NiFe_2_O_4_/GO for visualizing of elements arrangement in ZnS-chitosan.

Figure 11 displays the elemental mapping images obtained through EDX analysis. The spatial distribution of iron, cobalt, nickel, carbon, and oxygen exhibits a close match, indicating the colocalization of these elements. This finding suggests a homogeneous dispersion of MFe_2_O_4_ (M = Co, Ni) nanoparticles within the graphene oxide matrix, as evidenced by the uniform presence of particles throughout the mapped region. Furthermore, the mapping of carbon, nitrogen, and oxygen aligns with the uniform morphology of the composite. In summary, the EDX spectroscopy and mapping unequivocally confirm the successful integration of MFe_2_O_4_ nanoparticles into the graphene oxide, resulting in the desired nanocomposite structure.

### TGA-DTG analysis

TGA-DTG analysis is a powerful thermal analysis technique that combines thermogravimetric analysis (TGA) and differential thermal analysis (DTG). TGA involves measuring weight changes in a sample as it undergoes controlled temperature increases in a specific atmosphere. This provides valuable insights into the sample's thermal stability, decomposition behavior, phase transitions, and composition. DTG, used alongside TGA, measures the rate of temperature change over time or temperature, aiding in the identification of specific thermal events like endothermic or exothermic processes, phase transitions, and decomposition reactions. By integrating TGA and DTG, we can gain a comprehensive understanding of a sample's thermal behavior. TGA-DTG analysis is particularly useful for studying the thermal stability, degradation, and thermal events of various materials such as polymers, composites, minerals, pharmaceuticals, and organic compounds^24^.

**Figure 12:**
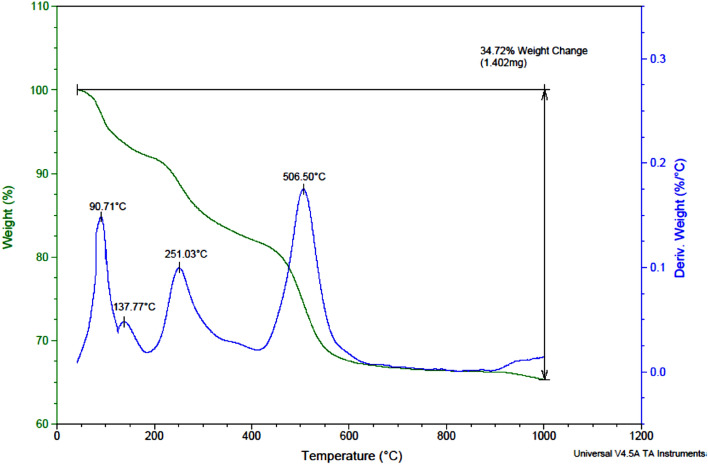
Thermogravimetric analysis curve of NiFe_2_O_4_/GO.

Figure 12 displays the TGA-DTG analysis results of NiFe_2_O_4_/GO. The analysis reveals important insights into the thermal behavior of the sample. At 90 °C, a process is observed, indicating an initial weight loss peak associated with releasing water molecules from the sample. This peak is typically attributed to the removal of physically adsorbed water. Another weight loss peak is observed at 135 °C, signifying the release of additional water molecules. This temperature range is commonly linked to the elimination of chemically bound water or water molecules present in hydrated compounds. Furthermore, at 251 °C, an endothermic process is evident, corresponding to the weight loss of graphene oxide fragments. When graphene oxide is subjected to heat, it undergoes thermal decomposition, liberating various volatile compounds, including water, carbon dioxide, and other organic fragments. In addition, a distinct endothermic weight loss peak is observed at 506 ºC during the DTG analysis. This peak is associated explicity with the weight loss of metallic fragments of NiFe_2_O_4_. This peak suggests that the analyzed sample contains a material incorporating metallic fragments such as NiFe_2_O_4_. Heating of NiFe_2_O_4_ sample leads to the thermal decomposition, resulting in the release of volatile metallic fragments and the observed weight loss peak.

### DSC-DTA analysis

DSC-DTA analysis is an integrated technique for studying the thermal properties and behavior of materials. It combines DSC, which measures heat flow during controlled temperature changes to detect phase transitions, crystallization, melting, and more, with DTA, which measures temperature differences between the sample and a reference material to identify thermal events like decomposition and reactions. The combination of DSC and DTA allows for simultaneous measurement of heat flow and temperature difference, providing complementary information. The resulting DSC-DTA curve shows peaks and valleys corresponding to different thermal events, enabling analysis of the sample's composition, purity, phase transitions, and thermal stability^25^.

**Figure 13:**
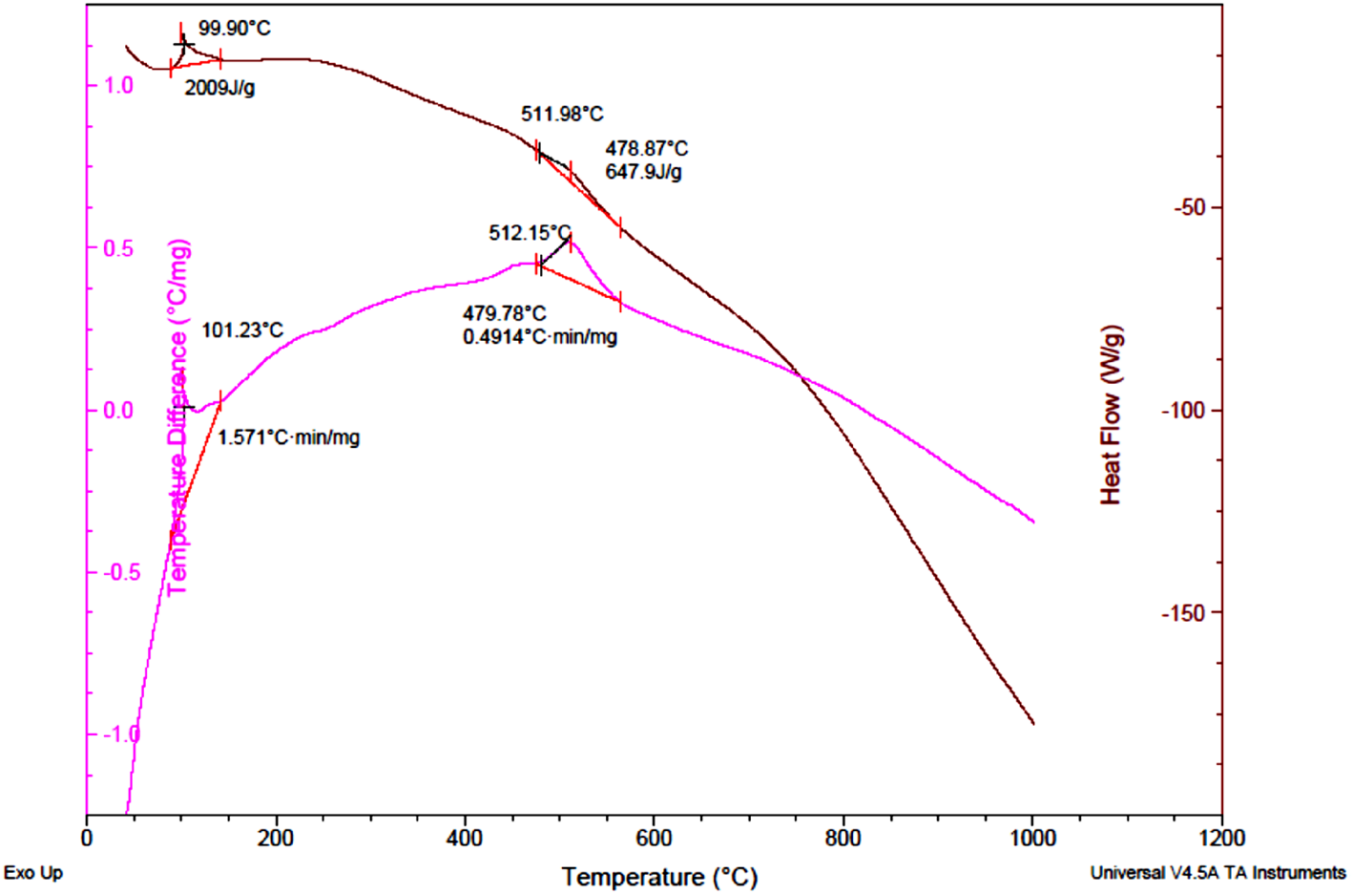
Differential scanning calorimetric curve of NiFe_2_O_4_/GO.

In Fig. 13, the DSC curves of NiFe_2_O_4_/GO within the temperature range of 40–1000 °C are presented. These curves reveal important thermal characteristics of the material. At around 99 °C, an endothermic peak is observed, indicating the dehydration of water molecules. The enthalpy required for releasing these water components was measured to be 2009J g^−1^. Additionally, another peak is observed at 512 °C, which is identified as an endothermic peak associated. This process includes the decomposition of graphene oxide. The enthalpy required for the release of graphene oxide fragment during this stage was determined to be 647.9 J g^−1^.

### BET analysis

**Figure 14:**
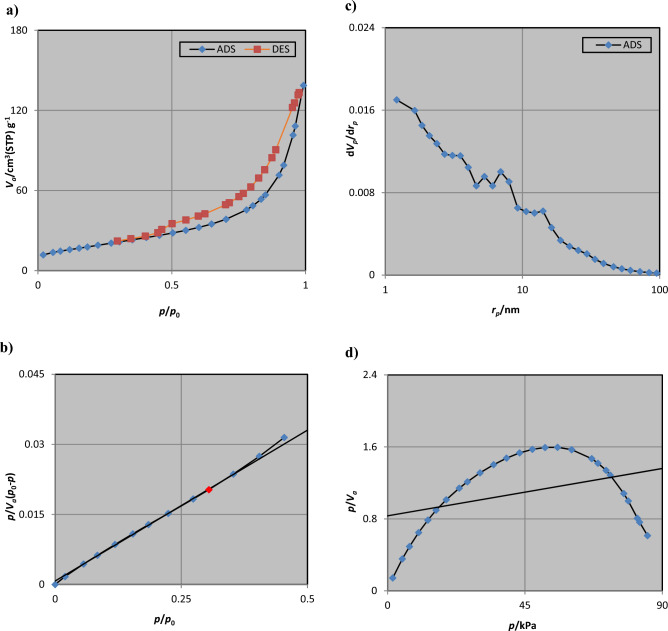
(**a**) Nitrogen adsorption–desorption isotherm, (**b**) BET analysis, (**c**) BJH curve, and (**d**) Langmuir plot for NiFe_2_O_4_/GO.

The surface area, average pore diameter, and pore volume of NiFe_2_O_4_/GO were determined using the BET technique. This involved investigating the volume of N_2_ adsorbed/desorbed in the pores as a function of relative partial pressure^26^. The nitrogen adsorption–desorption analysis was conducted at 77 K, and the corresponding results are depicted in Fig. 14.

The adsorption–desorption analysis of NiFe_2_O_4_/GO provides compelling evidence of a reduction in pore-specific surface area and pore volume upon intercalation of GO into the NiFe_2_O_4_ nanostructure. Table 3 displays the calculated fractional porosities using BJH desorption volume, as well as the corresponding values for surface area, pore size, and pore volume. Each pore exhibited a diameter of approximately 1.21 nm, with a mean pore diameter of 12.67 nm, which confirmed the mesoscopic nature of the nanocomposite. Furthermore, the Langmuir surface area and Vm were determined to be 744.36 m^2^g^−1^ and 171.02 cm^3^(STP)g^−1^, respectively.

**Table 3:** Values of pore size, surface area, and pore volume in BET, Langmuir, t, and BJH plots.

BET plot	
V_m_	15.299 cm^3^(STP) g^−1^
a_s,BET_	66.59 m^2^ g^−1^
*C*	88.265 cm^3^g^−1^
Total pore volume (*p*/*p*_0_ = 0.990)	0.02111 cm^3^ g^−1^
Mean pore diameter	12.679 nm
Langmuir plot	
V_m_	171.02 cm^3^(STP)g^−1^
a_s,Lang_	744.36 m^2^g^−1^
B	0.0070133
t plot	
Plot data	Adsorption branch
a_1_	29.929 m^2^ g^−1^
V_1_	0.010165 cm^3^ g^−1^
BJH plot	
Plot data	Adsorption branch
V_p_	0.2094 cm^3^ g^−1^
*r*_*p,peak*_ (Area)	1.21 nm
a_p_	70.149 m^2^ g^−1^

### Dye removal studies using CoFe_2_O_4_ and CoFe_2_O_4_/GO

**Figure 15:**
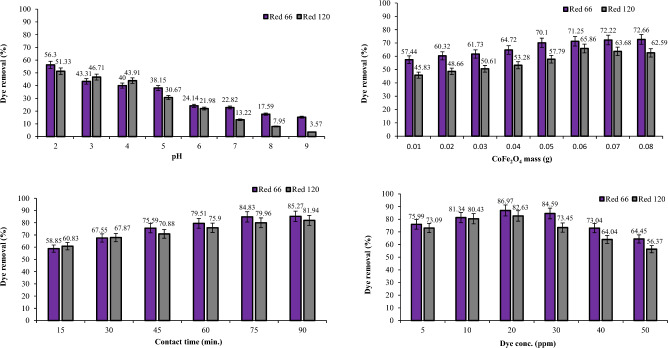
Effect of pH, dosage, dye concentration, and contact time on Red 66 and Red 120 removal using CoFe_2_O_4_.

**Figure 16:**
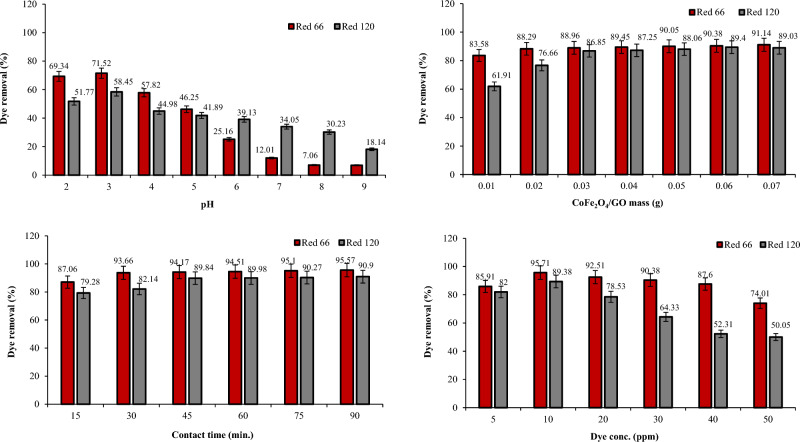
Effect of pH, dosage, dye concentration, and contact time on Red 66 and Red 120 removal using CoFe_2_O_4_/GO.

The removal efficiency of Red 66 and Red 120 dyes was investigated by employing CoFe_2_O_4_ and CoFe_2_O_4_/GO (Figs. 15 and 16). The experiments were conducted at different pH levels.

In acidic conditions, the presence of H^+^ ions becomes more abundant, causing an elevation in the surface charge of the photocatalyst^27–29^. At pH 2, CoFe_2_O_4_ exhibited a removal efficiency of 56.3% for Red 66 and 51.33% for Red 120. Furthermore, when CoFe_2_O_4_/GO was used at pH 2, the removal efficiency improved to 69.34% for Red 66 and 51.77% for Red 120. When the pH was increased to 3, there was a decrease in the removal efficiency of both dyes using CoFe_2_O_4_. Cobalt ferrite demonstrated a removal efficiency of 43.31% for Red 66 and 46.71% for Red 120 at this pH. However, it is important to note that utilizing CoFe_2_O_4_/GO resulted in significant improvements in dye removal. Specifically, the removal efficiency increased to 71.52% for Red 66 and 58.45% for Red 120 when CoFe_2_O_4_/GO was employed. As the pH was further increased to 4, a decline in the removal efficiency was observed for both CoFe_2_O_4_ and CoFe_2_O_4_/GO composites. Specifically, the removal efficiency of CoFe_2_O_4_ decreased to 40% for Red 66 and 57.81% for Red 120. Similarly, the removal efficiency of CoFe_2_O_4_/GO dropped to 43.91% for Red 66 and 44.98% for Red 120 at pH 4. Moreover, at pH 5, the removal efficiency of both CoFe_2_O_4_ and CoFe_2_O_4_/GO declined even further. As the pH continued to increase to 6, there was a notable decrease in the removal efficiency of both Red 66 and Red 120 when using CoFe_2_O_4_. Specifically, the removal efficiency dropped to 25.16% for Red 66 and 39.13% for Red 120. Similarly, when CoFe_2_O_4_/GO was employed, the removal efficiency values were 24.14% for Red 66 and 21.98% for Red 120 at pH 6. At pH 7, the removal efficiency decreased for both CoFe_2_O_4_ and CoFe_2_O_4_/GO composites. This indicates that higher pH levels negatively impact the removal efficiency of the composites. Furthermore, at pH 8, there was a significant reduction in the removal efficiency. CoFe_2_O_4_ exhibited removal efficiency values of 17.59% for Red 66 and 7.95% for Red 120, while CoFe_2_O_4_/GO showed values of 12.01% for Red 66 and 34.05% for Red 120. The results underscore the crucial role of pH in influencing the removal efficiency of Red 66 and Red 120 dyes, employing CoFe_2_O_4_ and CoFe_2_O_4_/GO composites. Notably, the maximum removal efficiency was observed at pH 3, indicating the significance of maintaining this specific pH for optimal dye removal. As the pH deviated from this optimal value, a decline in removal efficiency was observed. These findings emphasize the necessity of carefully adjusting the pH when utilizing CoFe_2_O_4_ and CoFe_2_O_4_/GO composites for the effective removal of Red 66 and Red 120 dyes from the solution. By optimizing the pH conditions, it is possible to enhance the removal efficiency and achieve more efficient and successful dye removal.

The experiment involved manipulating the mass of the CoFe_2_O_4_ and CoFe_2_O_4_/GO within a range of 0.01–0.07 g (Figs. 15 and 16). The CoFe_2_O_4_ nanocomposite demonstrated varying percentages of Red 66 dye removal, with 57.44, 60.32, 61.73, 64.72, 70.10, 71.25, 72.22, and 72.66%. For Red 120 removal using CoFe_2_O_4_, the recorded percentages were 45.83, 48.66, 50.61, 53.28, 57.79, 65.86, 63.68, and 62.59%. In contrast, the CoFe_2_O_4_/GO nanocomposite exhibited significantly higher percentages of dye removal. Precisely, for Red 66 removal, the percentages were measured at 83.58, 88.29, 88.96, 89.45, 90.05, 90.38, and 91.14%. Similarly, for Red 120 removal, the percentages were 61.91, 76.66, 86.85, 87.25, 88.06, 89.40, and 89.03%. These results indicate that the utilization of the CoFe_2_O_4_/GO nanocomposite resulted in substantially higher removal percentages for both Red 66 and Red 120 dyes compared to the CoFe_2_O_4_ nanocomposite alone. This highlights the enhanced performance and efficiency of the CoFe_2_O_4_/GO nanocomposite in removing these dyes.

The experiment investigated the impact of contact time on the removal of Red 66 and Red 120 dyes (Figs. 15 and 16). The contact time ranged from 15 to 90 min, and UV adsorption measurements were taken during each interval. During the initial 15-min sampling period, the CoFe_2_O_4_ nanocomposite achieved removal percentages of 58.85% for Red 66 and 60.83% for Red 120. However, the highest removal efficiencies for both dyes were observed after 90 min of reaction time when using CoFe_2_O_4_. Impressive removal percentages of 85.27% for Red 66 and 81.94% for Red 120 were achieved at this time point. In contrast, when utilizing the CoFe_2_O_4_/GO nanocomposite, the removal amounts were significantly higher during the same time frame. The CoFe_2_O_4_/GO nanocomposite achieved removal percentages of 95.57% for Red 66 and 90.9% for Red 120 after 90 min of contact time. These results demonstrate the superior performance of the CoFe_2_O_4_/GO nanocomposite compared to CoFe_2_O_4_ alone. Incorporating graphene oxide into the nanocomposite structure led to a substantial enhancement in the dye removal efficiency, with the peak performance observed at 90 min of contact time.

The experiment investigated the impact of different dye concentrations, specifically 5, 10, 20, 30, 40, and 50 ppm (Figs. 15 and 16). The highest percentages of Red 66 and Red 120 removal were observed when the dye concentration was set at 20 ppm for the CoFe_2_O_4_ nanocomposite. Remarkably, when using CoFe_2_O_4_/GO, an impressive dye removal of 95.71% for Red 66 and 89.38% for Red 120 was achieved at a dye concentration of 10 ppm under identical experimental conditions. These results highlight the significantly superior dye removal efficiency of the CoFe_2_O_4_/GO nanocomposite compared to CoFe_2_O_4_ alone. Incorporating graphene oxide into the nanocomposite structure led to a substantial enhancement in dye removal efficiency, even at lower dye concentrations.

### Dye removal studies using NiFe_2_O_4_ and NiFe_***2***_O_4_/GO

**Figure 17:**
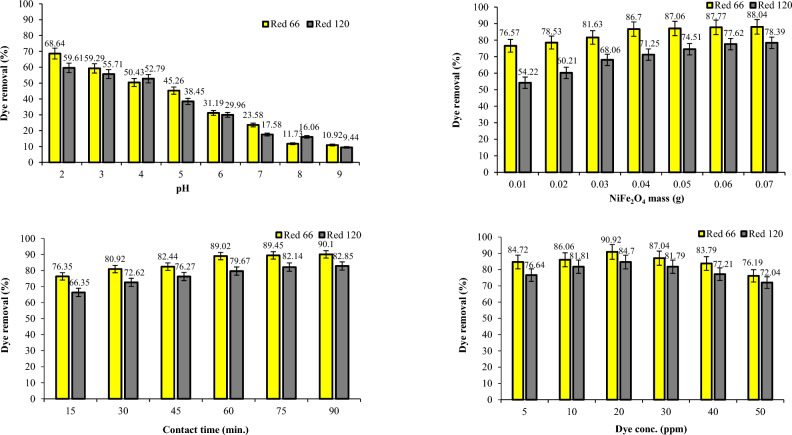
Effect of pH, dosage, dye concentration, and contact time on Red 66 and Red 120 removal using NiFe_2_O_4_.

**Figure 18:**
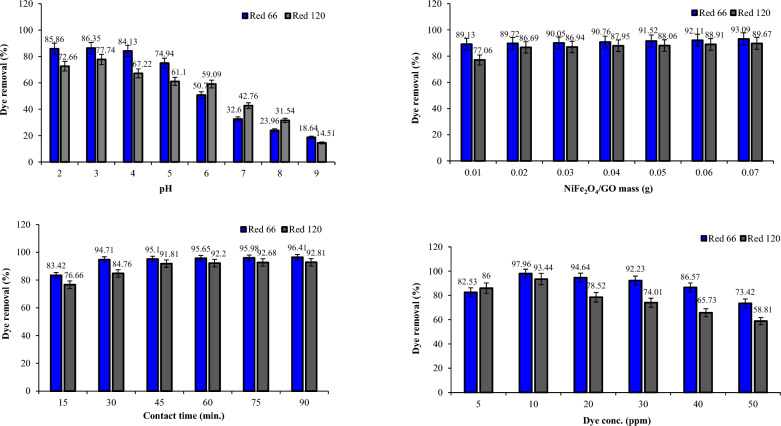
Effect of pH, dosage, dye concentration, and contact time on Red 66 and Red 120 removal using NiFe_2_O_4_/GO.

The removal efficiency of Red 66 and Red 120 dyes was investigated using NiFe_2_O_4_ and NiFe_2_O_4_/GO composites at varying pH levels (Figs. 17 and 18). Adding GO to NiFe_2_O_4_ improved the removal efficiency for both dyes compared to using NiFe_2_O_4_ alone. Specifically, at pH 2, NiFe_2_O_4_ exhibited 68.64% and 59.61% removal efficiency for Red 66 and Red 120 dyes, respectively. However, when NiFe_2_O_4_/GO was utilized at the same pH, the removal efficiency was enhanced to 85.86% for Red 66 and 72.66% for Red 120. Testing at pH 3 revealed NiFe_2_O_4_ removed 59.29% of Red 66 dye and 55.71% of Red 120 dye. Also, NiFe_2_O_4_/GO exhibited dye removal efficiencies of 86.35% for Red 66 and 77.74% for Red 120. Therefore, maximum removal efficiency was achieved at pH 2 and 3 for NiFe_2_O_4_ and NiFe_2_O_4_/GO, respectively. As the pH increased beyond 3, a decline in removal efficiency was observed for both NiFe_2_O_4_ and NiFe_2_O_4_/GO. The results demonstrate that pH has a significant influence on the removal efficiency when using these materials. Incorporating GO with NiFe_2_O_4_ leads to improved dye removal, especially at acidic pH levels. Careful control of pH is necessary to optimize the removal efficiency of Red 66 and Red 120 dyes when employing NiFe_2_O_4_ and NiFe_2_O_4_/GO composites.

Varying the mass of NiFe_2_O_4_ and NiFe_2_O_4_/GO from 0.01 to 0.07 g showed NiFe_2_O_4_/GO significantly increased dye removal percentages compared to NiFe_2_O_4_ alone (Figs. 17 and 18). For Red 66, NiFe_2_O_4_ removal ranged from 76.57 to 88.04%, while NiFe_2_O_4_/GO removal was 89.13–93.09%. For Red 120, NiFe_2_O_4_ removal was 54.22–78.39%, versus 77.06–89.67% with NiFe_2_O_4_/GO. The results demonstrate the addition of GO to NiFe_2_O_4_ dramatically enhances its ability to remove both dyes. NiFe_2_O_4_/GO exhibited substantially higher removal percentages, highlighting its superior performance for dye removal.

Varying contact times from 15 to 90 min revealed the impact on Red 66 and Red 120 removal by NiFe_2_O_4_ and NiFe_2_O_4_/GO (Figs. 17 and 18). With NiFe_2_O_4_, removal was 76.35 and 66.35% for Red 66 and Red 120 at 15 min, increasing to 90.10 and 82.85% at 90 min. However, NiFe_2_O_4_/GO achieved significantly higher removal, 96.41% for Red 66 and 92.81% for Red 120 after 90 min. The results show NiFe_2_O_4_/GO greatly outperformed NiFe_2_O_4_, with optimal removal occurring at 90 min and incorporating graphene oxide enhanced efficiency substantially.

Changing the dye concentration from 5 to 50 ppm showed the impact on Red 66 and Red 120 removal by NiFe_2_O_4_ and NiFe_2_O_4_/GO (Figs. 17 and 18). NiFe_2_O_4_ achieved maximum removal at 20 ppm for both dyes (90.92 and 84.70%). However, with NiFe_2_O_4_/GO, 97.96 and 93.44% of Red 66 and Red 120 were removed at just 10 ppm. The results demonstrate NiFe_2_O_4_/GO's superior efficiency, removing more dye at lower concentrations, and incorporating graphene oxide significantly enhanced performance. NiFe_2_O_4_/GO showed substantially higher removal, highlighting improved efficiency even at reduced dye levels.

**Figure 19:**
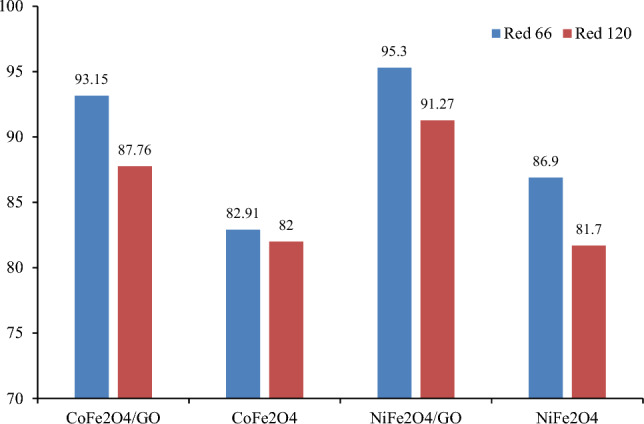
Dye removal (%) using CoFe_2_O_4_, NiFe_2_O_4_, CoFe_2_O_4_/GO, and NiFe_2_O_4_/GO under sun light irradiation.

Additional experiments measured dye removal using CoFe_2_O_4_, NiFe_2_O_4_, CoFe_2_O_4_/GO, and NiFe_2_O_4_/GO under sunlight irradiation for three days (Fig. 19). The results showed NiFe_2_O_4_/GO composite had higher photodegradation and improved dye removal compared to CoFe_2_O_4_/GO. The presence of NiFe_2_O_4_ on the GO surface enhanced the photodegradation process.

### Kinetic studies

The adsorption efficiency can be evaluated by employing kinetic models, such as the pseudo-first-order and pseudo-second-order equations. The pseudo-first-order kinetic model is commonly utilized to describe dye adsorption. Equation 1 represents this model:

1 $${\text{ln}}\left( {{\text{q}}_{{\text{e}}} - {\text{q}}_{{\text{t}}} } \right) = {\text{lnq}}_{{\text{e}}} - {\text{k}}_{{1}} {\text{t}}$$

In Eq. (1), q_e_ and q_t_ represent the adsorption capacity at equilibrium and at different times, respectively. The rate constant of the adsorption process's pseudo-first-order model is denoted by k_1_ (min^−1^).

The pseudo-second-order model identifies chemisorption as the rate-limiting step, where adsorption can occur at sites where no interactions between the adsorbates take place. The equation representing this model is expressed in Eq. (2):

2 $${\text{t}}/{\text{q}}_{{\text{t}}} = {1}/{\text{k}}_{{2}} {\text{q}}_{{{\text{eq}}}}^{2} + {\text{t}}/{\text{q}}_{{{\text{eq}}}}$$

In Eq. (2), k_2_ (g∙mg^−1^∙min^−1^) is the rate constant of pseudo-second-order adsorption.

**Figure 20:**
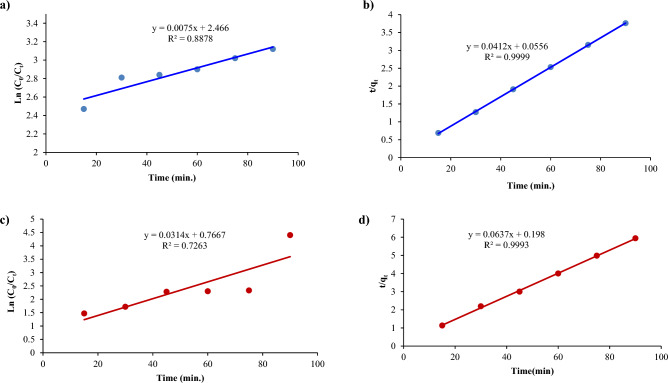
Kinetic studies of (**a**, **c**) Pseudo-first-order and (**b**, **d**) Pseudo-second order using CoFe_2_O_4_ on Red 66 (**a**, **b**) and Red 120 (**c**, **d**) dyes.

**Figure 21:**
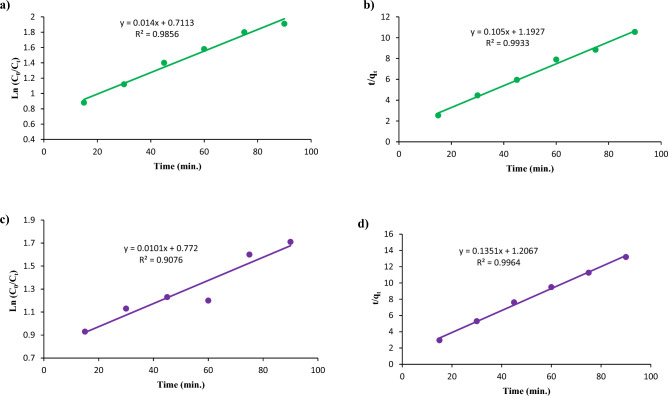
Kinetic studies of (**a**, **c**) Pseudo-first-order and (**b**, **d**) Pseudo-second order using CoFe_2_O_4_/GO on Red 66 (**a**, **b**) and Red 120 (**c**, **d**) dyes.

For CoFe_2_O_4_ on Red 66 and Red 120 removal, the parameters obtained from the pseudo-first-order model have R^2^ values of 0.88 and 0.72, respectively. On the other hand, the R^2^ values for the second-order kinetic model using CoFe_2_O_4_ are 0.99 and 0.99 (Fig. 20). When using CoFe_2_O_4_/GO, the pseudo-first-order model yielded parameters with R^2^ values of 0.98 and 0.90 for the removal of Red 66 and Red 120, respectively. In contrast, the second-order kinetic model using CoFe_2_O_4_/GO produced R^2^ values of 0.99 and 0.99, as shown in Fig. 21.

**Figure 22:**
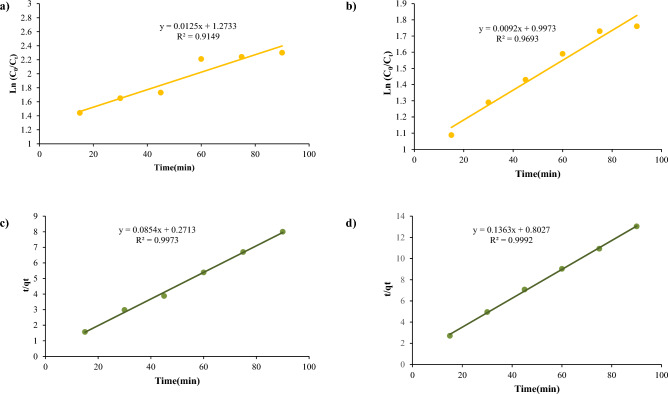
Kinetic studies of (**a**, **c**) Pseudo-first-order and (b, d) Pseudo-second order using NiFe_2_O_4_ on Red 66 (**a**, **b**) and Red 120 (**c**, **d**) dyes.

**Figure 23:**
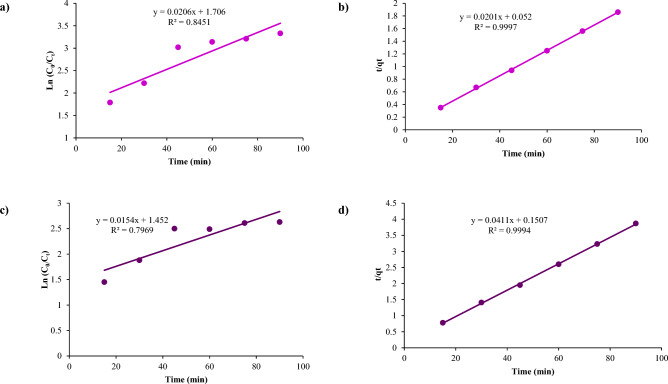
Kinetic studies of (**a**, **c**) Pseudo-first-order and (**b**, **d**) Pseudo-second order using NiFe_2_O_4_/GO on Red 66 (**a**, **b**) and Red 120 (**c**, **d**) dyes.

Kinetic modeling showed the second-order model better fit the NiFe_2_O_4_ and NiFe_2_O_4_/GO removal data for both dyes (Figs. 22 and 23). For NiFe_2_O_4_, R^2^ values were 0.91 and 0.99 for Red 66 and Red 120 with the pseudo-first-order model versus 0.96 and 0.99 with the second-order model. Similarly, NiFe_2_O_4_/GO yielded pseudo-first-order R^2^ values of 0.84 and 0.79, while the second-order model gave 0.99 and 0.99. The higher R^2^ values indicate the second-order model more accurately describes the adsorption kinetics.

Based on these results, it can be concluded that the pseudo-first-order model is not highly accurate. The pseudo-second-order model, on the other hand, yields the most optimal outcomes and has been observed to be effective for anionic dyes.

### Dye removal from textile wastewater

The textile wastewater was filtered and treated photocatalytically under optimal conditions to produce a colorless solution. To determine initial and final dye concentrations, standard solutions were prepared by mixing R66 and wastewater. Varying volumes of R66 standard were added to flasks filled with 25 mL wastewater. Absorbance was measured and a calibration curve plotted. The concentration of the unknown sample was calculated from the curve equation by setting y = 0. NiFe_2_O_4_/GO achieved 90% removal efficiency for textile wastewater dye, indicating successful photocatalytic treatment.

## Conclusions

The findings of this study provide important insights into the photocatalytic degradation of Reactive Red 66 and Reactive Red 120 dyes using spinel ferrite nanoparticles and their graphene oxide composites. Our researcher group successfully synthesized and characterized spinel ferrite nanoparticles MFe_2_O_4_ (M = Co, Ni) and their nanocomposites with graphene oxide (MFe_2_O_4_/GO) using various techniques such as FT-IR, Raman, XRD, zeta potential, VSM, SEM/EDX. The analysis confirmed the successful formation of the desired materials. The addition of graphene oxide to MFe_2_O_4_ to create MFe_2_O_4_/GO nanocomposites resulted in improved photocatalytic performance and enhanced dye removal efficiency compared to the pure spinel ferrite nanoparticles. This suggests a synergistic effect when combining MFe_2_O_4_ with graphene oxide. Among the tested photocatalysts, the NiFe_2_O_4_/GO nanocomposite exhibited the highest photocatalytic degradation and removal efficiency for both Reactive Red 66 and Reactive Red 120 dyes under UV light. This nanocomposite achieved dye removal percentages of up to 97.96% and 93.44%, respectively. Our researcher group optimized key parameters such as pH, catalyst dosage, dye concentration, and light exposure time to maximize dye removal. They found that acidic pH, higher catalyst loading, lower dye concentrations, and longer irradiation favored higher removal percentages. Kinetic studies indicated that the pseudo-second order model provided a better fit for the adsorption data, with higher R^2^ values, suggesting that chemisorption was the rate-controlling step. Under sunlight exposure, the nanocomposites demonstrated excellent natural light activity and maintained high dye removal efficiency even after three days. Among the nanocomposites tested, NiFe_2_O_4_/GO exhibited the best performance. Moreover, the nanocomposites effectively eliminated dyes from textile wastewater samples, indicating their potential for practical applications in wastewater treatment. In conclusion, the prepared spinel ferrite/graphene oxide nanocomposites exhibited excellent photocatalytic performance under both UV and sun light. The results emphasize their potential as efficient photocatalysts for the removal of anionic dyes from wastewater.

## Data Availability

The datasets used and/or analyzed during the current study available from the corresponding author on reasonable request.
